# Emerging biology of noncoding RNAs in malaria parasites

**DOI:** 10.1371/journal.ppat.1010600

**Published:** 2022-07-07

**Authors:** Karina Simantov, Manish Goyal, Ron Dzikowski

**Affiliations:** Department of Microbiology & Molecular Genetics, The Kuvin Center for the Study of Infectious and Tropical Diseases, IMRIC, The Hebrew University-Hadassah Medical School, Jerusalem, Israel; Joan and Sanford I Weill Medical College of Cornell University, UNITED STATES

## Abstract

In eukaryotic organisms, noncoding RNAs (ncRNAs) have been implicated as important regulators of multifaceted biological processes, including transcriptional, posttranscriptional, and epigenetic regulation of gene expression. In recent years, it is becoming clear that protozoan parasites encode diverse ncRNA transcripts; however, little is known about their cellular functions. Recent advances in high-throughput “omic” studies identified many novel long ncRNAs (lncRNAs) in apicomplexan parasites, some of which undergo splicing, polyadenylation, and encode small proteins. To date, only a few of them are characterized, leaving a big gap in our understanding regarding their origin, mode of action, and functions in parasite biology. In this review, we focus on lncRNAs of the human malaria parasite *Plasmodium falciparum* and highlight their cellular functions and possible mechanisms of action.

## Introduction

Apicomplexan parasites comprise a large and diverse group of unicellular eukaryotic organisms responsible for devastating diseases in humans and animals [1]. *Plasmodium falciparum* is a member of this phylum, which is responsible for the deadliest form of human malaria [2,3]. Malaria parasites are estimated to infect approximately 200 to 250 million people annually worldwide, resulting in over half a million deaths, primarily of young children and pregnant women [2].

*P*. *falciparum* possesses a complex life cycle with distinct morphological and developmental stages that alternate between the human and mosquito hosts [4–7]. The progression of the parasite’s life cycle is accompanied by dynamic gene expression, in accordance with the developmental stage and environmental factors [8–10]. When taking into consideration the complexity of the parasite’s life cycle, one would expect these complexities to be attributed to a large genome with many genes and well-defined transcription factors. However, surprisingly, *Plasmodium* spp. roughly encode approximately 5,400 genes from a very compact genome (approximately 23 Mb) and lack many sequence-specific transcription factors [9,11–14].

Studies indicate that *Plasmodium* parasites have evolved several distinctive complementary molecular mechanisms that act either at the transcriptional or posttranscriptional level to fine-tune the parasite’s RNA and protein repertoires at different stages of its life cycle [8,15–18]. These include but are not limited to, the presence of atypical transcription factors, alternative splicing (AS) and polyadenylation, mRNA export and degradation machinery, chromatin remodeling and epigenetic modifications, translational repression, and regulatory noncoding RNAs (ncRNAs) [16,17,19–24]. The presence of numerous putative RNA binding proteins (RBPs) in the *Plasmodium* genome further supports the idea that RNA metabolism plays an important role in gene regulation in these parasites [25,26]. Over the past decade, with the advances in the technology of whole-genome transcriptomics, it became apparent that *Plasmodium* parasites express numerous noncoding transcripts, which include long ncRNAs (lncRNAs) and circular RNAs (circRNAs) [21,26–35]. In addition, it was recently discovered that putative small proteins (<100 aa) are translated from lncRNAs [35]; however, very little is known about their function. The presence of numerous ncRNAs molecules was also reported from additional protozoan parasites including *Toxoplasma gondii*, *Cryptosporidium parvum*, *Leishmania* spp., *Giardia lamblia*, and *Trypanosoma* spp. [36–41]. In higher eukaryotes, ncRNAs are known as regulators of transcription and translation [42], RNA splicing [43], chromosome structure [44], and were even implicated in hormone-like activities [45]. However, the functions of only a limited number of noncoding transcripts have been characterized so far in apicomplexan parasites.

Here, we address the recent advance in the knowledge of some of the best-characterized lncRNAs in *P*. *falciparum* and discuss their unique characteristics in the parasite’s biology. We highlight how lncRNAs serve key regulatory roles, with a specific focus on mechanistic and functional paradigms. A better understanding of regulatory ncRNAs will provide us with a new perspective on the complexity of the gene regulatory network not only in *Plasmodium* but possibly also in additional apicomplexan parasites.

### lncRNAs: What do we know in *Plasmodium*?

#### Types of ncRNAs in *Plasmodium*

Generally, ncRNAs can be divided into 2 main groups: small noncoding RNAs (sncRNAs) and lncRNAs. sncRNAs comprise a wide variety of small RNAs (approximately 18 to 200 nt), including microRNAs (miRNAs), small interfering RNAs (siRNAs), and PIWI (P-element induced wimpy testis)-interacting RNAs (piRNAs) [46]. siRNA and miRNA are part of the RNA interference (RNAi) process where they regulate the degradation of transcripts mediated by the RNA-induced silencing complex (RISC). They target various transcripts for silencing by guiding RISC to the target mRNA through complementary base-pairing. The target mRNAs are subsequently degraded by the RISC-associated argonaute (AGO) RNase. siRNAs differ from miRNAs by their origin, where siRNA is derived from long double-strand RNA (dsRNA) and miRNA is generated from hairpin-shaped precursors [47,48]. As a result, the level of complementation and subsequent targeting of mRNAs for degradation differs between the 2 sncRNAs. miRNAs have a 5′ seed region that can target multiple mRNAs through partial complementation, whereas siRNAs regulate specific mRNAs that fully complement their sequence [46–48]. Since its discovery, the RNAi machinery is widely used to achieve an inducible and reversible knockdown of a specific gene in eukaryotic cells. miRNAs and components of RISC have been identified in apicomplexan parasites such as *T*. *gondii* [49,50], however, it appears that the RNAi pathway is different than other eukaryotes [51]. In marked contrast, *Plasmodium* parasites lack most of the components of the RNAi pathway rendering them RNAi-deficient organisms, which could explain the lack of endogenous miRNAs in the parasite [52,53].

**Fig 1:**
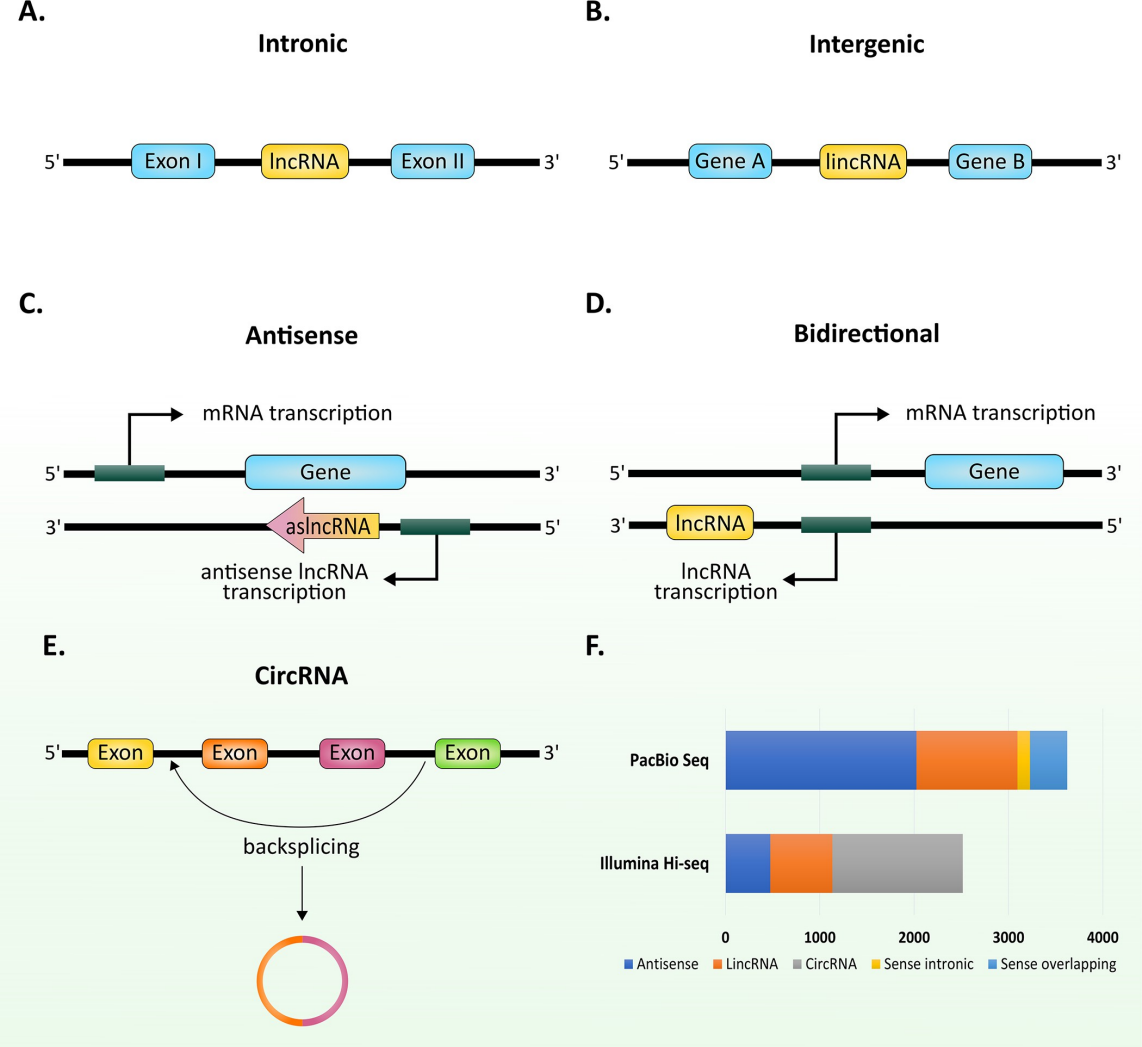
Graphical representation of different types of lncRNAs identified in *P*. *falciparum*. (A) Intronic lncRNAs, transcribed from the intronic region of protein-coding genes. (B) lincRNA, located between 2 protein-coding genes. (C) Antisense lncRNAs, transcribed from the antisense strand of protein-coding genes. (D) Bidirectional lncRNA, transcribed from the opposite strand by a bidirectional promoter, and (E) circRNAs generated through back-splicing of transcripts with intronic, exonic or intronic and exonic fragments. (F) Putative lncRNA identified in *P*. *falciparum* using PacBio and Illumina sequencing [27,35]. Both studies reported a relatively large number of differentially expressed lncRNAs during intra erythrocytic development. circRNA, circular RNA; lncRNA, long ncRNA; lincRNA, long intergenic lncRNA.

lncRNAs (>200 nt) are usually transcribed by RNA polymerase II and were shown to be involved in various cellular processes such as transcription, chromosome remodeling, and protein trafficking [54,55]. lncRNA are classified according to their position into 5 main groups: (I) sense lncRNA; (II) intronic lncRNAs; (III) antisense lncRNAs; (IV) bidirectional lncRNAs; and (V) long intergenic ncRNA (lincRNA) (Fig 1A–1E) [56,57]. Since their discovery, extensive studies on the functions of lncRNAs have uncovered their crucial regulatory roles in many organisms. Renowned examples include regulation of X chromosome inactivation in female mammalian cells through the lincRNA *Xist* [58], recruitment of histone-modifying enzymes to gene promotors, such as the promotor-associated antisense lncRNA *Airn* in mice that regulates *Igf2r* gene silencing [59], functioning as a scaffold that interacts with multiple proteins or complexes, such as the *HOTAIR* lncRNA transcribed from the HOXC gene cluster [60].

#### The growing plethora of ncRNAs in *Plasmodium* parasites

With the advance in our understanding of the regulatory nature of ncRNAs in eukaryotes, efforts to identify ncRNAs in *Plasmodium* spp. have greatly accelerated. However, most studies have been focused on *P*. *falciparum*, leaving much to be discovered in other species. The completion of *P*. *falciparum’s* genome sequencing and assembly enabled the annotation and further characterization of coding and noncoding elements. Early transcriptomic studies using northern blots, microarray, and serial analysis of gene expression (SAGE) revealed the widespread prevalence of stage-specific antisense transcripts throughout the *Plasmodium* genome [21,28,31,33,61–65]. Additionally, computational predictions and structural analyses of the noncoding genome assisted in the identification of several candidate ncRNAs and novel structured RNAs [30,31,63,66–68]. In recent years with the advances in sequencing technologies that facilitated accurate strand-specific, direct single-molecule pore-sequencing [69] as well as single-molecule real-time (SMRT) full-length sequencing, it became apparent that *P*. *falciparu*m transcribes over ≥2,500 full-length lncRNAs, including lincRNAs, natural antisense transcripts (NATs), sense-overlapping and sense-intronic ncRNAs transcripts, as well as over 1,300 circRNAs [27,29,32,35,69] (Fig 1F and Table 1). It is important to note that the observed large number of ncRNAs in *Plasmodium*, an organism with a compact genome and high gene densities, could be due to overlapping/fused mRNA transcripts and transcriptional byproducts. Therefore, it will be important to authenticate these transcriptomic results with functional assays.

**Table 1:** Various sequencing techniques used to study ncRNAs in *P*. *falciparum*.

Sequencing	Output	Advantage	Disadvantage	Reference
cDNA and EST database analysis	630 novel ncRNAs	• Early tool for transcript structure and variants analysis • Produced increasingly accurate gene models	• Labor-intensive and time-consuming • Shows inherent clone bias • Incomplete sequence coverage	[31]
Next-generation Illumina sequencing (RNA-seq)	977 new splice sites, 310 AS events, and splicing of antisense transcripts	• Less time consuming compared to cDNA clone/ESTs sequencing	• Difficult to identify complex AS events, full-length splicing isoforms, and other transcripts (coding and noncoding) • Lack of strand specificity	[21]
Strand-specific RNA-seq	201 new AS events, 660 lincRNA, 474 NATs, and 1,381 circRNAs	• Differentiation between antisense and sense transcripts	• Similar challenges as short-read RNA-seq	[27,32]
Oxford Nanopore Technologies (ONT), direct single molecule pore-sequencing	Full length reads of 1,835 genes, 1,112 AS events, intron retained accounts for 60% to 68% of the total AS genes	• Direct RNA-seq approach • Simple sample preparation • Long reads, easy to assemble, high coverage • Potential identification of nucleotide modification • Straightforward antisense transcripts recognition	• Relatively high base calling error rates • Sequencing depth • Long run time	[69]
Pacific Biosciences (PacBio) SMRT full-length sequencing	393 AS events, 3,623 lncRNAs, 1,555 APA events, 1,721 fusion transcripts	• Simple preparation and fast run time • Long reads, easy to assemble, high coverage • Simple differentiation between sense and antisense transcripts	• Indirect sequencing • Relatively high error rates • Number of reads per run	[35]

APA, alternative polyadenylation tag; AS, alternative splicing; circRNA, circular RNA; EST, expressed sequence tag; NAT, natural antisense transcript; lincRNA, long intergenic noncoding RNA; lncRNA, long noncoding RNA; SMRT, single-molecule real-time; UTR, untranslated region.

Interestingly, the expression of numerous antisense transcripts from multiple sites across the genome was found to vary depending on the parasite stage, the gene locus, the culture conditions, and the parasite line being investigated [31,32,61,62,64,65,70,71]. Moreover, the expression timing of NATs and their neighboring mRNAs could be either inversely or positively correlated [62,65]. It would be interesting to determine whether these variable expression profiles are linked with different cellular functions and regulatory mechanisms. Altogether, these studies indicate that *P*. *falciparum* harbors a wide variety of ncRNAs; however, the function of most of its ncRNA is yet to be discovered.

#### Functionally characterized lncRNAs in *Plasmodium*

**Fig 2:**
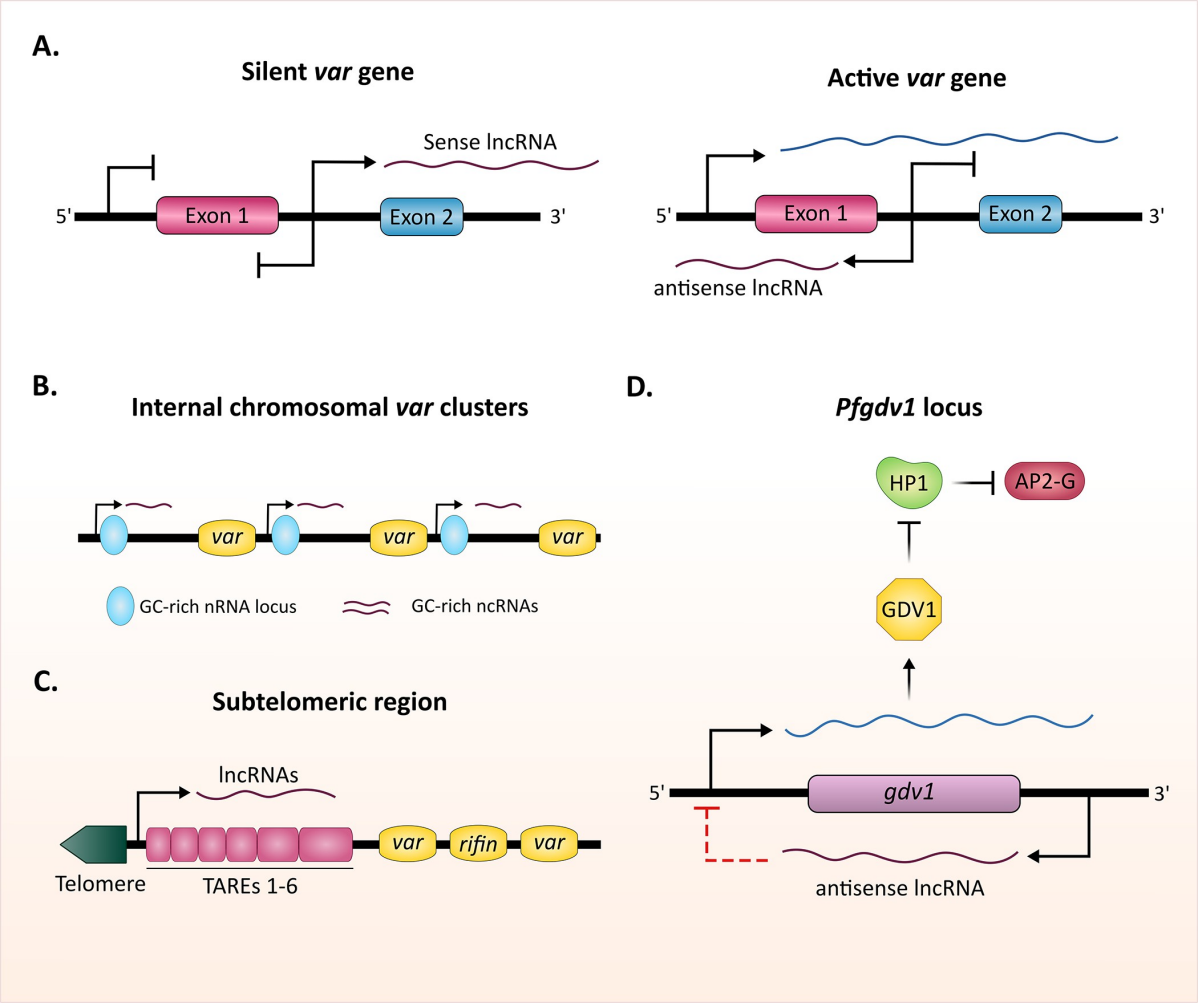
Functionally characterized lncRNAs in *P*. *falciparum*. (A) lncRNAs involved in mutually exclusive expression of *var* genes. Schematic representation of a *var* gene locus composed of a variable exon 1, bidirectional promoter within the intron, and a conserved exon 2. The upstream *var* promoter engages in the transcription of the mRNA, while the intronic bidirectional promoter is involved in the production of sense and antisense lncRNA transcripts. The silent *var* gene transcribes sense ncRNA from the promoter within the intron (left). In the active state of the *var* gene, RNA polymerase II transcribes both an mRNA in the sense direction and an lncRNA in the antisense direction (right). (B) Intergenic GC-rich ncRNAs transcribed from internal *var* chromosomal clusters in *P*. *falciparum*. These GC-rich ncRNAs are predicted to be transcribed by RNA polymerase III. (C) Schematic representation of *P*. *falciparum* telomere and TAREs and subtelomeric gene families. (D) lncRNA’s involvement in sexual commitment. Sexual commitment in *Plasmodium* is regulated by GDV1, whose expression is antagonistically regulated by its antisense lncRNA. Once expressed, GDV1 reverses HP1-dependent silencing of AP2-G and inhibits sexual commitment. GDV1, gametocyte development 1; HP1, heterochromatin protein 1; lncRNA, long ncRNA; ncRNA, noncoding RNA; TARE, telomere-associated repeat.

Nonetheless, over the last decade, several noncoding transcripts have been characterized and implicated as regulators of key biological processes in the parasite. A prominent example is the lncRNAs involved in the regulation of expression of PfEMP1 (*P*. *falciparum* erythrocyte membrane protein 1) [72–74]. These variable surface proteins are the major ligands responsible for *P*. *falciparum’s* pathogenicity and its ability to evade human immunity. Immune evasion is achieved in part by cytoadherence of the infected red blood cells (iRBCs) by attachment of PfEMP1 to several endothelial receptors. Consequently, the iRBC is removed from the circulation and avoids clearance by the spleen. Thus, cytoadherence and sequestration of iRBCs are the main cause of tissue damage and the severe pathogenicity during *P*. *falciparum* malaria [73,74]. In addition, the parasites have evolved a unique antigenic switching mechanism to avoid the antibody-mediated response against PfEMP1. This is achieved by switches in expression among a multicopy gene family named *var*, where each *var* gene encodes for a different PfEMP1 variant and is expressed in a mutually exclusive manner [72–76]. Immune evasion through antigenic switching between different PfEMP1 variants depends on the parasite’s ability to tightly regulate and ensure that only a single *var* gene is expressed at a time, and to be able to switch the expression to a different *var* gene as the antibody-mediated response develops. The *var* gene structure includes a variable exon 1, a conserved intron with a bidirectional promoter, and a conserved exon 2 [77,78]. The bidirectional intron promoter transcribes 2 lncRNAs, which were implicated in regulation of antigenic switching [79–83]. The first is a sense lncRNA that extends into the conserved second exon, and the second is an antisense lncRNA complementary to the 3′ of the first exon (Fig 2A) [73]. These lncRNAs are transcribed by RNA Pol II, undergo capping, but are not polyadenylated [71]. They localize to distinct perinuclear foci in the nucleus and are incorporated into the chromatin [78]. While the sense *var* lncRNA appears to be transcribed from all the *var* genes and accumulates during the late stages of the parasite’s development [77,84], the antisense lncRNA is expressed only from the single active *var* gene at the early stage of the parasite’s intraerythrocytic development (IDC) when *var* mRNA is transcribed (Fig 2A) [73]. To date, no functional role has been assigned to the *var* sense lncRNA; however, its accumulation during late stages when *var* genes are poised for transcription, and the fact that it is transcribed from all the *var* genes may imply that these transcripts could play a role in silencing, imprinting of the *var* gene family as a genetic unit for coordinated regulation of mutually exclusive expression, and potentially as regulators of epigenetic memory. On the other hand, the antisense *var* lncRNAs appeared to play a role in the activation of the single *var* gene transcribed, though the exact mechanism of action is still not clearly understood [72,78,79,85]. It was found that the expression of specific antisense lncRNAs in *trans* can activate a silent *var* gene in a sequence and dose-dependent manner. In addition, interfering with the antisense lncRNA of an active chromosomal *var* gene leads to the down-regulation in its expression and alters its epigenetic imprint, which results in switching in expression to different *var* genes [72,86]. Interestingly, the antisense lncRNAs were also associated with the active *var* gene when an exonuclease termed PfRNAse II was down-regulated [87]. It has also been reported that the disruption of PfRNAse II’s function by fusing it with a destabilization domain led to the dual expression of 2 different *var* genes, both expressing their respective antisense lncRNAs. This observation led to the hypothesis that PfRNAse II could potentially be involved in the targeted degradation of antisense lncRNAs of a specific *var* subtype, and thus contribute to their regulation [87].

In addition to these *var* lncRNAs transcribed by each *var* gene, additional ncRNAs were discovered within intergenic regions near *var* genes located within internal chromosomal clusters. Interestingly, these ncRNAs have a high GC-content that is uncommon in the *P*. *falciparum* genome that is extremely AT-rich (approximately 80%), particularly in the intergenic regions (Fig 2B) [13,34,66,88,89]. The sequence of these GC-rich ncRNAs is relatively conserved and contains elements corresponding with RNA polymerase III promoter, which may imply that the transcription of the GC-rich ncRNAs is mediated by RNA Pol III, unlike other *var* lncRNAs transcribed by RNA Pol II [34]. Furthermore, it appears that their expression is clonally variable where different clonal parasite populations express different transcripts [89]. However, while 15 GC-rich ncRNAs were found in the 3D7 genome, this parasite line encodes for approximately 60 *var* genes. Nonetheless, these GC-rich ncRNAs were found to colocalize with the expression sites of subtelomeric and internal *var* genes and their overexpression was shown to cause de-repression of a subset of *var* genes [89]. A recent study used a CRISPR interference (CRISPRi) strategy to target the entire GC-rich repertoire by guiding dCas9 to the conserved DNA sequence found in all the transcripts. Down-regulation of the GC-rich transcripts led to a corresponding down-regulation of several multicopy gene families including *var*, *rifin*, *stevor*, and *Pfmc-2TM* [88], suggesting that the expression of the GC-rich ncRNAs is involved in the transcriptional activity of these gene families. It is still unclear whether these conserved transcripts are involved in the choice for activation of the single *var* gene expressed, particularly since their sequence, as well as the effect of their down-regulation, appears not to be *var* specific. It is possible that they play a role in maintaining the chromosomal configuration that positions active genes in subnuclear foci that enable transcriptional activity. Intriguingly, these GC-rich transcripts were also shown to act as repressors in *cis*, possibly by acting as insulator elements that influence the spread of heterochromatin [34]. It will be important to understand the mechanism that orchestrates this dual, and potentially conflicting activities, of transcriptional silencing and activation of these regulators.

Another subclass of lncRNAs identified in *P*. *falciparum* are telomeric and subtelomeric associated lncRNAs, transcribed from the telomere-associated repetitive elements (TAREs) during the late stages of IDC (Fig 2C) [27,28,90,91]. The TAREs consist of 6 repetitive blocks (TAREs 1 to 6) that vary in their length and DNA sequence. These repetitive elements are located between the telomere and the coding region of the first subtelomeric genes, where TARE-1 is closest to the telomere end, and TARE-6 is adjacent to the first coding region [90]. The TARE-lncRNAs can be subclassified into 2 main groups. The first is a lncRNA of approximately 4 kb long, derived from the region from TARE-3 to the telomere (TARE-3-lncRNA). The second is a longer transcript over 6 kb long, derived from TARE-6 (TARE-6-lncRNA) (Fig 2C) [28,91]. During the early stages of IDC, these TARE-lncRNAs localize to a single perinuclear compartment (with unknown function), whereas during the late stages they localize to several foci on the nuclear periphery [91]. The sequences of the TARE-lncRNAs appear to be enriched with binding sites for various transcription factors, supporting their possible involvement in the regulation of neighboring genes. They were also postulated to be involved in the maintenance of the structural integrity of the telomere and of chromosome ends [27,28,91]. These hypotheses are supported by the structure of TARE-6-lncRNA that is comprised of 21 bp repeats that form secondary hairpin structures that can bind histones and other nuclear proteins. In addition, TARE-6-lncRNA was implicated in regulating chromosomal conformation and heterochromatin structure during the late stages of IDC [91]. A strand-specific RNA-seq study linked the expression of TARE-lncRNAs with the timing of *var* sterile lncRNAs, suggesting a possible joined mechanism between these 2 types of lncRNAs [27].

An additional lncRNA transcript assigned a biological function in *P*. *falciparum* is the *gdv1* lncRNA implicated to be involved in the regulation of sexual development. Sexual commitment in *P*. *falciparum* is triggered by the master transcriptional regulator PfAP2-G (Fig 2D) [92]. In asexual blood-stage parasites, *Pfap2-g* is silenced by heterochromatin protein 1 (PfHP1) [93,94]. However, during sexual commitment, *P*. *falciparum* gametocyte development 1 protein (PfGDV1) displaces PfHP1 from the *Pfap2-g* locus [95]. Removal of PfHP1 from the locus changes chromatin conformation to open euchromatin that facilitates *Pfap2-g* transcription and sexual conversion. Interestingly, this study found that the *Pfgdv1* gene transcribes an antisense lncRNA that negatively regulates the expression of *Pfgdv1*. Thus, the antisense *Pfgdv1* lncRNA functions as an inhibitor of sexual differentiation (Fig 2D) [95].

**Fig 3:**
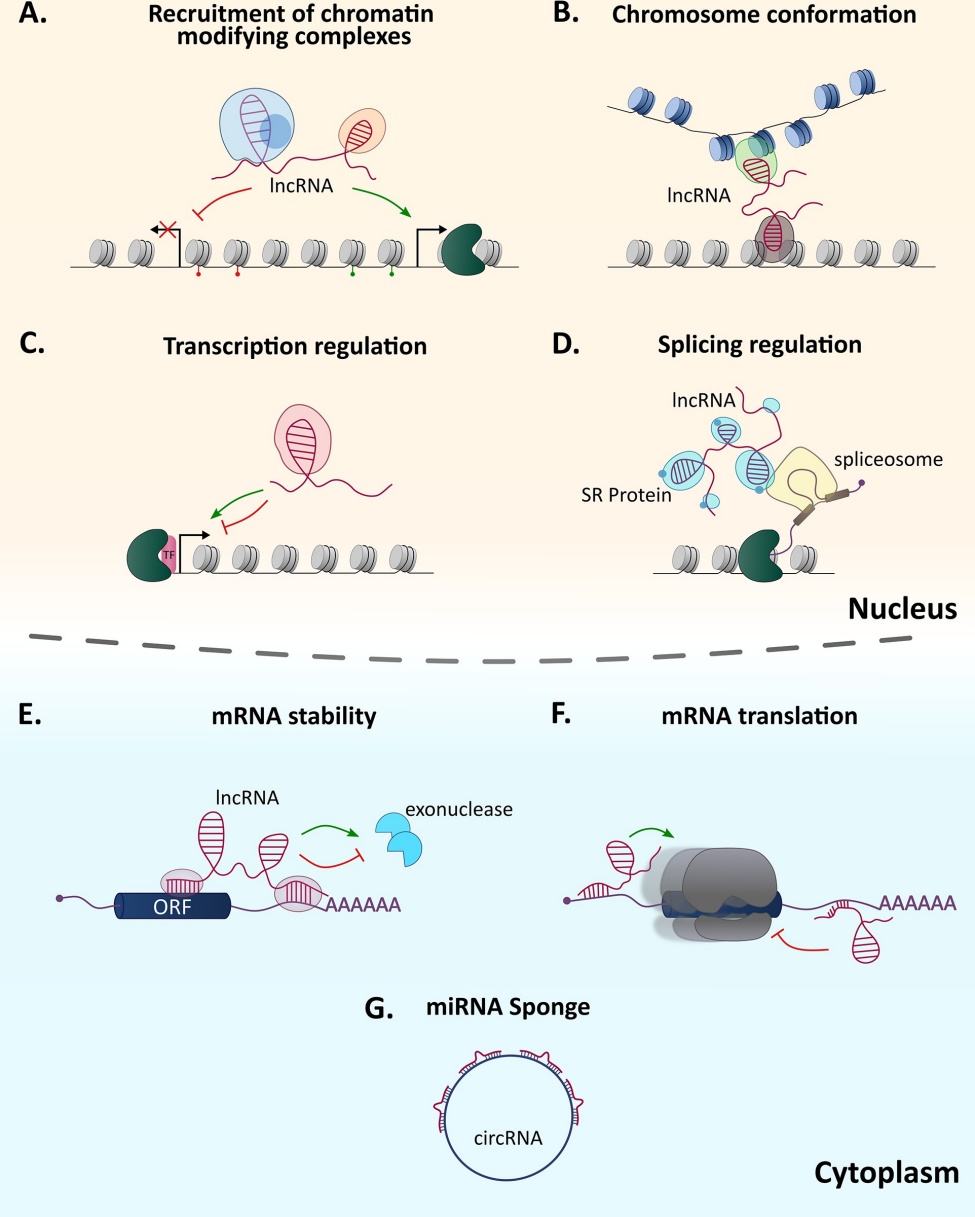
lncRNAs operate by different mechanisms. The subcellular localization of lncRNAs determines their mechanisms and biological functions. Inside the nucleus (upper) they can perform regulatory functions such as (A) scaffolds for recruitment of regulatory factors and protein complexes, (B) regulation of chromatin conformation/chromatin architecture, (C) transcription regulation of target genes, and (D) regulation of pre-mRNA splicing. Cytoplasmic lncRNAs (lower) are involved in the modulation of (E) mRNA stability and decay, (F) mRNA translation, and act as a (G) miRNA sponge to inhibit host (human)-encoded miRNAs. circRNA, circular RNA; lncRNA, long ncRNA; miRNA, microRNA.

circRNAs are additional unique forms of lncRNAs that were identified in *P*. *falciparum*. Strikingly, hundreds of unknown circRNAs were discovered using advanced strand-specific RNA-seq [27]. Out of the putative circRNAs identified, only a small subset was longer than 200 bp. These indications were validated by divergent PCR across the predicted splice junction on 6 of the long circRNAs [27]. circRNAs are thought to function as competitive inhibitors by binding miRNAs like a sponge, leading to a reduction in the miRNA pool that is available to bind target mRNAs (Figs 1E and 3G) [96,97]. However, since *P*. *falciparum* does not encode for miRNAs or the RNAi machinery [52,53], it seems unlikely that these circRNAs encoded by *P*. *falciparum* function as miRNA sponges in the parasite. Intriguingly, some of the long circRNAs contain predicted binding sites for human miRNAs, and one can speculate that these circRNAs could be involved in regulatory mechanisms of host–parasite interactions. The presence of human erythrocytic miRNAs was reported in parasites during the IDC, and some of these miRNAs were shown to cause a reduction in parasite’s proliferation in culture, while others were implicated in interfering with *var* gene expression [98–100]. These findings along with changes in the levels of human miRNAs found in iRBCs [101–103] suggest that some *P*. *falciparum* ncRNAs had evolved to interact with human miRNAs to manipulate their function and maintain infection.

Taken together, the discovery of such a large repertoire of lncRNAs in *P*. *falciparum* highlights the shortcomings in our knowledge regarding the function of these noncoding transcripts in the molecular mechanisms that regulate gene expression which enable the parasite to thrive with such a complex cell cycle.

## How do lncRNAs regulate gene expression?

lncRNAs were implicated in several layers of gene regulation, including chromatin organization, transcription, splicing, RNA stability, and translation (Fig 3) [104–107]. To account for such functional diversity, different lncRNAs were shown to operate in several molecular mechanisms. For example, lncRNAs can act as scaffolds that organize chromosomal structures and guide different macromolecular complexes to assemble in a specific subcellular compartment to work together. They can also act as decoys and block certain molecular pathways by binding and inhibiting the function of proteins. In addition, lncRNAs were suggested to act as signal molecules that function as regulators that mediate the transcription of downstream gene subsets [104]. While these mechanisms have been extensively studied and characterized for lncRNAs in other organisms, their exact mode of action in *P*. *falciparum* is unknown.

Many lncRNAs function in the nucleus where they regulate gene expression and modify the chromatin structure through crosstalk with epigenetic factors [104–107]. Some lncRNAs recruit chromatin-modifying complexes to specific genomic loci, leading to changes in the compactness of the chromatin, which in turn results in the up- or down-regulation of gene transcription (Fig 3A) [106,107]. Additionally, lncRNAs can regulate the spatial conformation of chromosomes by functioning as a scaffold and mediating interchromosomal interactions at specific genomic loci that can facilitate interactions between long distanced enhancers/silencers with specific promoters (Fig 3B) [108]. lncRNAs can also regulate transcription by interacting and recruiting transcription factors and/or components of the transcriptional machinery to activate/silence genes [109]. Therefore, lncRNAs could regulate transcription in *cis* or in *trans*, i.e., in proximity or further away from their transcription site (Fig 3C) [55]. It is reasonable to hypothesize that such mechanisms could explain the function of the *var* antisense lncRNA in *P*. *falciparum*. One could speculate that the *var*-specific antisense lncRNA may be involved in the recruitment of chromatin-modifying enzymes or other factors that can facilitate the transcriptional activation of a single *var* promoter (Fig 2A). Furthermore, the act of RNA Pol II transcription per se of a specific lncRNA can epigenetically imprint the locus and its surrounding area for activation, resulting in the transcriptional activation of nearby genes [109]. Similar to higher eukaryotes, it was suggested that in *P*. *falciparum*, the phosphorylation pattern of the C terminal domain (CTD) of RNA Pol II determines the appropriate recruitment of the various factors that coordinate precise transcription, as well as epigenetic memory and imprinting [110]. Interestingly, the histone methyltransferase PfSET2 that directly binds the CTD is recruited to *var* gene loci, marking them with H3K36me3. This histone modification appears to be an epigenetic recognition marker for multicopy gene families (and TAREs) and is not found in other gene loci transcribed by RNA Pol II [86,111]. This restricted distribution pattern appears to be unique to *P*. *falciparum* in comparison to other eukaryotes [86,112]. It was proposed that this specificity is regulated by the phosphorylation patterns of the Pol II CTD during ncRNA transcription, which differs from those patterns during pre-mRNA transcription, explaining how PfSET2 recruitment is restricted to these loci [110,111]. It is tempting to suggest that the ncRNAs themselves might serve as molecular signals that may influence the specific CTD phosphorylation pattern.

In addition to these possible mechanisms, the act of lncRNA transcription could have a silencing effect, where the active transcription of an lncRNA interferes or blocks the transcription machinery of a nearby gene [113,114]. Such collision could explain the inhibition of PfGDV1 expression when its antisense lncRNA is transcribed. However, it is also possible that the antisense lncRNA of *Pfgdv1* functions by different mechanisms and regulates RNA stability and/or translation.

In other organisms, telomeric lncRNAs termed TERRA (telomeric repeat-containing RNA) are implicated in the maintenance of telomeres and telomerase function [115,116]. They were found to interact with various telomere-associated proteins (such as HP1, the origin recognition complex (ORC), and components of the Shelterin complex) to establish and maintain heterochromatin at the telomere ends [117,118]. Interestingly, TERRA also interacts with RAD51 during R-loop formation at chromosome ends promoting homology-dependent recombination (HDR) [118,119]. The TARE-lncRNAs transcribed by *P*. *falciparum* near members of virulent multigene families such as *stevor*, *rifin*, and *var* share many similarities with TERRA lncRNAs. TARE-lncRNAs were implicated in regulating the structural integrity of the telomeres and the genomic loci of these virulent genes, thus affecting their expression (Fig 2C) [90,120]. It was proposed that TARE-lncRNAs could be involved in recruitment of chromatin regulators such as PfOrc1, PfSir2, and PfKMT1 to these subtelomeric regions. In addition, they could be involved in heterochromatin formation at subtelomeric loci, through interaction with the major heterochromatic hallmarks of *P*. *falciparum*, H3K9me3, and PfHP1 [85,94,121–123]. Another possible similarity is the involvement of PfRad51 in telomere healing used by the parasites to repair double-stranded breaks within the subtelomeric regions [124,125]. One can speculate that TARE-lncRNAs may play a role in this process by interacting with PfRAD51, like TERRA-lncRNAs. Together, these similarities raise new hypotheses and possibilities for further research toward understanding these mechanisms.

At the posttranscriptional level, lncRNAs function as modulators of mRNA splicing, stability, and translation (Fig 3D–3F). Their interactions with RBPs and their ability to bind to complementary sequences of targeted mRNAs enable them to regulate these processes in a sequence-specific manner [126]. mRNA splicing is a complex process regulated by the binding of many RBPs and splicing factors. It has been postulated that NATs may influence the splicing pattern of pre-mRNAs by their association with the transcript, creating RNA-RNA duplex structures that in turn can result in masking of splice sites or by inhibition of spliceosome recruitment and/or function [127]. Other lncRNAs were found to regulate splicing by binding to splicing factors such as SR proteins and by modulating their distribution and activity (Fig 3D). For example, the lncRNA MALAT1 regulates a pool of active serine-arginine (SR) proteins by sequestering them to nuclear speckle domains until they are needed [128]. In *P*. *falciparum*, splicing of pre-mRNAs is an important step in mRNA maturation, particularly because over half of the parasite’s genes contain introns [13,24]. Moreover, the evolutionary conservation of this process further highlights the importance of splicing in the parasites. While the mechanism of splicing and the various splicing factors involved is still largely unknown, studies have found several conserved splicing and alternative factors [21,24,66,129–131]. Therefore, it would be interesting to investigate the possible involvement of lncRNAs in regulation of constitutive and AS in *Plasmodium* spp.

The regulation of mRNA stability, turnover, and translation in the cytoplasm are the final regulatory layers of gene expression. These interconnected processes directly affect the level of protein production and were found to be regulated by RBPs and cytoplasmic lncRNAs [132–134]. lncRNAs can affect the function of RBPs by promoting their recruitment or dissociation from target mRNAs thereby influencing mRNA decay of certain transcripts (Fig 3E). In addition, lncRNAs can interact with translation initiation factors or ribosome-associated proteins to regulate translation (Fig 3F) [126,133,135]. Furthermore, lncRNAs were found to regulate these processes by influencing the levels and patterns of RNA modifications such as m^6^A. Consequently, the secondary structure of the mRNA transcripts could change and alter their interaction with RBPs or other regulatory factors, resulting in changes in their translation levels [132,136]. *P*. *falciparum* contains an overrepresentation of RBPs and surprisingly high levels of highly structured mRNAs that could interact with lncRNAs to regulate mRNA stability and translation rate [137,138]. Furthermore, the levels of the m^6^A modification in *P*. *falciparum* exceed those found in any other eukaryotes and are developmentally regulated [139]. Considering the interplay between the secondary structure and the dynamics of the m^6^A modification, one can question whether lncRNAs in *P*. *falciparum* play a role in regulating the rates of m^6^A levels and thus affect mRNA stability during the IDC.

Unveiling the specific interactions of lncRNAs with either DNA sequences, other transcripts, and/or proteins may provide important indications for their possible cellular function. Therefore, implementing novel methodologies such as ChIRP-seq [140] and RADICL-seq [141] together with ChIRP-MS [142] and CLIP [105,143] could be utilized to uncover their functions in *P*. *falciparum*.

## Are all lncRNAs noncoding?

**Fig 4:**
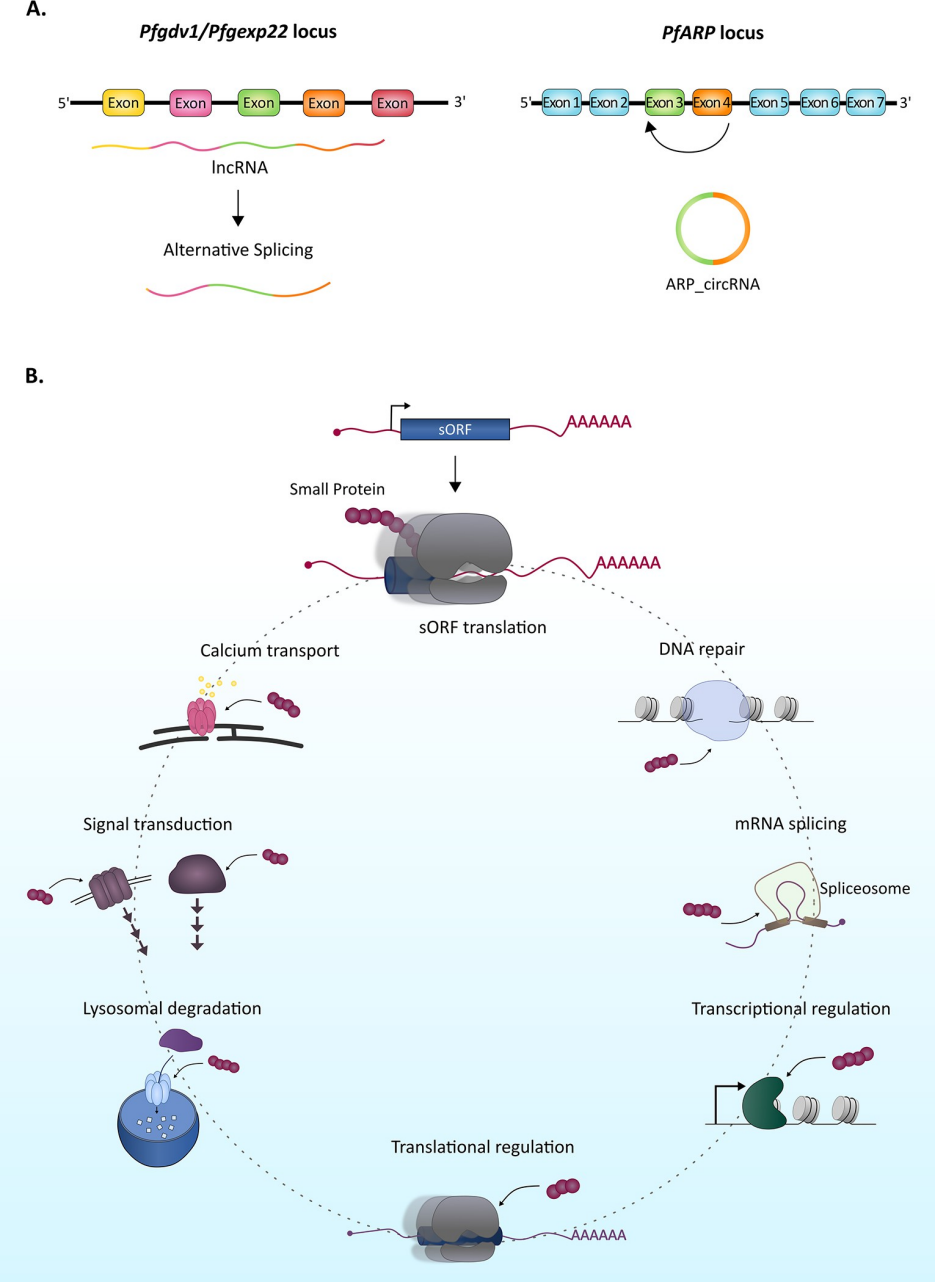
Splicing and translation of lncRNAs. (A) *P*. *falciparum* lncRNAs can undergo AS (left panel) and generate circRNAs as demonstrated for ARP_circRNA (apoptosis related protein circRNA) (right panel). (B) Some lncRNAs contain sORFs that are translated into small proteins with different biological roles, such as transcriptional and translational regulation, mRNA splicing, signal transduction, mitochondrial regulation, calcium transport, and lysosomal degradation. AS, alternative splicing; circRNA, circular RNA; lncRNA, long ncRNA; sORF, small open reading frame.

Historically, lncRNAs were designated as RNA transcripts that are not spliced and do not contain open reading frames that are translated to proteins [54,144,145]. Intriguingly, recent advances in computational capabilities and molecular techniques (such as more precise mass spectrometry, deep RNA sequencing, and ribosome profiling) have revealed that certain lncRNAs undergo posttranscriptional processing and are implicated in several human diseases [146,147]. Similar to mRNAs, lncRNAs may contain exons and introns, undergo constitutive and AS, interact with ribosomes, and contain small open reading frames (sORFs) that could encode for small proteins [148–150] (Fig 4). These findings imply that lncRNAs are a potential source for an unexplored repertoire of ncRNAs and small proteins [151,152].

lncRNAs that undergo AS produce novel RNA sequences that could either function as ncRNAs or protein-coding transcripts [146,151,153]. Numerous AS events comprising approximately 5% of the total transcripts were reported in *P*. *falciparum*, including a few examples for splicing of lncRNAs, such as GEXP22 lincRNA, PfGDV1 antisense lncRNA, and antisense transcripts (Fig 4A, left) [21,27,29,130]. Additionally, the detection of numerous circRNAs in *P*. *falciparum* further supports splicing of lncRNAs, since their circular structure is generated by splicing as demonstrated for the ARP_circRNA (Fig 4A, right) [27]. Nonetheless, compared to other eukaryotic organisms, our knowledge of AS in *P*. *falciparum* is restricted, primarily due to the limitations in identifying these events.

Transcriptomic analyses and ribosome profiling experiments have shown that many lncRNA transcripts are associated with ribosomes and contain sORFs [146–148,150,153]. Numerous lncRNA-encoded small proteins (<100 aa), previously overlooked due to their small size, have been identified in evolutionary distinct organisms from bacteria to high eukaryotes (Fig 4B) [148,150]. Furthermore, some of these small proteins are predicted to be highly conserved among different species [148,154,155]. The expression of the lncRNA-encoded small proteins could be highly specific to a certain cell type, tissue, and developmental stage, indicating their possible specialized cellular functions. Interestingly, some lncRNAs, such as the SRA (steroid receptor RNA activator), function as noncoding transcripts in the nucleus, and in addition to their nuclear function, they can get spliced, exported, and translated to small proteins in the cytoplasm [156]. However, the factors that control this interchangeable function are unknown.

LncRNA-encoded small proteins have been shown to be involved in diverse cellular processes ranging from transcriptional regulation to cell division, DNA repair, and signaling pathways (Fig 4B) [154,157–163]. Most notable examples include bacterial MciZ that regulates cell division [160], bacterial Sda involved in regulation of signal transduction [158], Scl involved in calcium metabolism and cardiac contraction in *Drosophila* [162], hemotin in *Drosophila* implicated in regulation of endosomal maturation during phagocytosis [154], mitoregulin (Mtln) involved in mitochondrial complexes and respiratory efficiency in mouse and human [163], myoregulin (MLN) that regulates muscle performance in mouse and human [157], and human MRI-2 involved in the end joining DNA repair pathway [164].

Approximately 160 small proteins corresponding to sORF regions of lncRNAs were recently identified in *P*. *falciparum* at different stages of IDC [35]. In addition, circRNAs, which appear to be abundant in *Plasmodium*, were also demonstrated to have the capacity to encode small proteins in vitro and in vivo in other organisms [165–167]. However, their biological activities and protein-coding capabilities in *Plasmodium* remain to be uncovered [27]. Considering the emerging importance of lncRNAs-encoded small proteins, it is reasonable to hypothesize that implementing state of the art methodologies such as ribosome profiling, poly-Ribo-Seq, and proteogenomic methodologies followed by reverse genetic approaches will lead to the identification and characterization of a novel subset of small proteins with fundamental biological functions in *Plasmodium*.

### Concluding remarks

Evidently, regardless of their protein-coding purpose, many RNA molecules have different intrinsic regulatory functions. In the last couple of decades, numerous discoveries shed light on the important role of lncRNAs as regulators of cellular processes in many organisms. A relatively rich plethora of ncRNAs are transcribed by *Plasmodium* spp., but surprisingly, very little is known about their biological functions and even less about the molecular and cellular mechanisms by which they act. Nonetheless, several ncRNAs were implicated in fundamental aspects of the parasite’s biology, such as regulation of antigenic variation and sexual commitment, emphasizing their potential roles as emerging key regulators of various biological processes in apicomplexan parasites and other eukaryotes.
